# Spiritual care needs and their attributes among Chinese inpatients with advanced breast cancer based on the Kano model: a descriptive cross-sectional study

**DOI:** 10.1186/s12904-024-01377-8

**Published:** 2024-02-22

**Authors:** Zhangyi Wang, Xiaochun Tang, Liping Li, Huifang Zhou, Yue Zhu, Lamei Chen, Tao Su, Mengru Liu, Xiaoli Pang, Xiaoke Yi, Li Liu, Jingjing Liu, Mengsu Liu

**Affiliations:** 1 Nursing Department, Central Hospital of Hengyang, No.10, Yancheng Road, Yanfeng District, Hengyang, 421001 Hunan China; 2 School of Nursing, Tianjin University of Traditional Chinese Medicine, Tianjin, China; 3 Kidney Transplantation Department, Shandong Provincial Hospital, Shandong First Medical University, Jinan, Shandong China; 4 Blood Purification Center, The Second Affiliated Hospital of University of South China, Hengyang, Hunan China

**Keywords:** Spiritual care needs, Attributes, Advanced breast cancer, Inpatients, Kano model

## Abstract

**Background:**

Numerous previous research have established the need for spiritual care among patients with cancer globally. Nevertheless, there was limited research, primarily qualitative, on the spiritual care needs of Chinese inpatients with advanced breast cancer. Furthermore, the need for spiritual care was rarely explored using the Kano model. To better understand the spiritual care needs and attributes characteristics of inpatients with advanced breast cancer, this study examined the Kano model.

**Methods:**

A descriptive cross-sectional design study was conducted in the oncology departments of three tertiary grade-A hospitals in China from October 2022 to May 2023. To guarantee high-quality reporting of the study, the Strengthening the Reporting of Observational Studies in Epidemiology Checklist was used. Data on the demographic characteristics questionnaire, the Nurse Spiritual Therapeutics Scale (NSTS), and the Kano model-based Nurse Spiritual Therapeutics Attributes Scale (K-NSTAs) were collected through convenience sampling. The Kano model, descriptive statistics, two independent samples *t*-tests, and one-way analysis of variance were used to analyze the data.

**Results:**

The overall score for spiritual care needs was 31.16 ± 7.85. The two dimensions with the highest average scores, “create a good atmosphere” (3.16 ± 0.95), and the lowest average scores, “help religious practice” (1.72 ± 0.73). The 12 items were distributed as follows: three attractive attributes were located in Reserving Area IV; five one-dimensional attributes were distributed as follows: three one-dimensional attributes were located in Predominance Area I, and two were found in Improving Area II; two must-be attributes were located in Improving Area II; and two indifference attributes were located in Secondary Improving Area III.

**Conclusion:**

The Chinese inpatients with advanced breast cancer had a middle level of spiritual care needs, which need to be further improved. Spiritual care needs attributes were defined, sorted, categorized, and optimized accurately and perfectly by the Kano model. And “create a good atmosphere” and “share self-perception” were primarily one-dimensional and must-be attributes. In contrast, the items in the dimensions of “share self-perception” and “help thinking” were principally attractive attributes. Nursing administrators are advised to optimize attractive attributes and transform indifference attributes by consolidating must-be and one-dimensional attributes, which will enable them to take targeted spiritual care measures based on each patient’s characteristics and unique personality traits.

**Supplementary Information:**

The online version contains supplementary material available at 10.1186/s12904-024-01377-8.

## Background

Worldwide, breast cancer is the most common cancer to affect women. Globally, the incidence of breast cancer is 2.9 million, which accounts for 31.0% of cancer incidence in women and 15.0% of all cancer-related deaths, according to the 2023 Global Cancer Statistics Report [1]. In China, there are 420,000 new cases of breast cancer, which ranks first in the country and accounts for 19.9% of all female cancer cases [2]. The majority of these cases are advanced forms of the disease. Surgery, radiotherapy, chemotherapy, targeted therapy, etc., are the hallmarks of breast cancer care. There will inevitably be side effects from one or more combinations of diagnosis and treatment plans, and the lengthier treatment cycle results in numerous health problems and negatively impacts the quality of life [3, 4]. During treatment, the patient will have significant social, psychological, and spiritual challenges related to altered body image, role changes, transformed sexual life, and fear of cancer recurrence [5]. Patients are particularly more severe if they have advanced breast cancer [6]. Therefore, spiritual care is necessary for patients with advanced breast cancer for them to overcome pain; appreciate the value of life; and find spiritual sustenance, gain hope, love, forgiveness, and strength, that is spiritual care needs [7, 8]. Furthermore, several research studies have demonstrated the potential benefits of spirituality in cancer coping strategies, diagnosis, treatment, and rehabilitation for patients with breast cancer. Moreover, spirituality is crucial to patient care and overall health advancement [9, 10].

The word “spiritus” in Latin, which means “breathing” and denotes an essential aspect of life, is where spirituality originated. People use it as a means of experiencing themselves, the present, other people, the ultimate, and the natural environment, and discovering and expressing life’s meaning and purpose [11]. According to many academics, everyone desires and expects to find the purpose and meaning of life and the relationship between themselves, others, God/holiness, faith, and nature. Although this urge may be connected to religion, people without religious views can also experience it [12, 13]. The importance of attending to the spiritual care needs of patients with cancer and offering them spiritual care has been confirmed globally. Many studies have demonstrated that spiritual care for patients with advanced cancer can help relieve pain and discomfort, build strong interpersonal relationships, receive emotional support, allay inner fears, promote rehabilitation, extend life expectancy, and enhance nursing quality and satisfaction [14–16]. These evaluation results of spiritual care needs and spiritual troubles also support the conclusions drawn from these studies. Chinese policymakers have acknowledged spiritual care, as evidenced by the 2017 publication of the Health Planning Commission of China issued by the Practice Guide for Hospice Care (Trial) [17], which said that hospice treatment should involve offering spiritual care to patients. However, patients with breast cancer may have more significant needs for spiritual care because of the influence of the prognosis and course of treatment of the disease.

According to a literature review, research on the spiritual care needs of patients with breast cancer overseas has been increasing annually due to the increasing incidence and mortality of the disease and its significance. Numerous studies have confirmed that patients with breast cancer require spiritual care. Devi et al.’s qualitative study [10] revealed that among Singapore’s newly diagnosed patients with breast cancer, transcending experience, meaning and purpose, and shifting perspectives are typical manifestations of spiritual care needs. Additionally, a different qualitative study revealed that the spiritual satisfaction of patients with breast cancer was associated with a sense of life value, a sense of community, and a natural connection [18]. Vilalta et al. [19] also discovered that the three most urgent spiritual care needs of patients with cancer are knowledge of the illness, respect, and belief. Furthermore, according to Lynn et al.’s qualitative research [20], patients with breast cancer believe that spirituality and religion—which includes attending religious ceremonies, praying, worshiping, and reading the Bible—can give them strength and encouragement to deal with the pain associated with the diagnosis and treatment of the disease. According to Agli et al. [21], religious beliefs may have a biased influence on the spiritual care needs of patients with cancer. According to Ghahramanian et al. [22], the majority of patients with cancer need spiritual care in the form of “thinking about and believing in God,” “being prayed for by others,” and “needing kindness and helping others.” Park et al. [23] showed how the spirituality of breast cancer survivors might help patients adopt good habits in several ways, which will improve their overall health. Finally, based on their respective cultural backgrounds, Fallah et al. [24] and Jafar et al. [25] implemented spiritual care interventions for patients with breast cancer. They discovered that incorporating spiritual care into group psychological interventions can significantly enhance the happiness, hope, quality of life, and satisfaction of patients with breast cancer.

Simultaneously, several qualitative studies conducted in China have demonstrated that patients with breast cancer have extensive spiritual care needs. These needs include a wide range of themes, including rediscovering life’s purpose, enhancing one’s sense of dignity, and taking on new roles [26, 27]. However, research on the spiritual care needs of Chinese inpatients with advanced breast cancer is still in its early stages. Furthermore, certain limitations are applied to the domestic study. First, few studies have used the Kano model to qualitatively analyze the attributes characteristics of spiritual care needs among patients with advanced breast cancer. Instead, the majority of research content in the literature has focused on the status quo and factors that influence spiritual care needs among patients with cancer. Second, the study design is relatively straightforward, and the majority of the qualitative research and the majority of the quantitative research in the previous Chinese studies were conducted using this methodology.

**Fig. 1:**
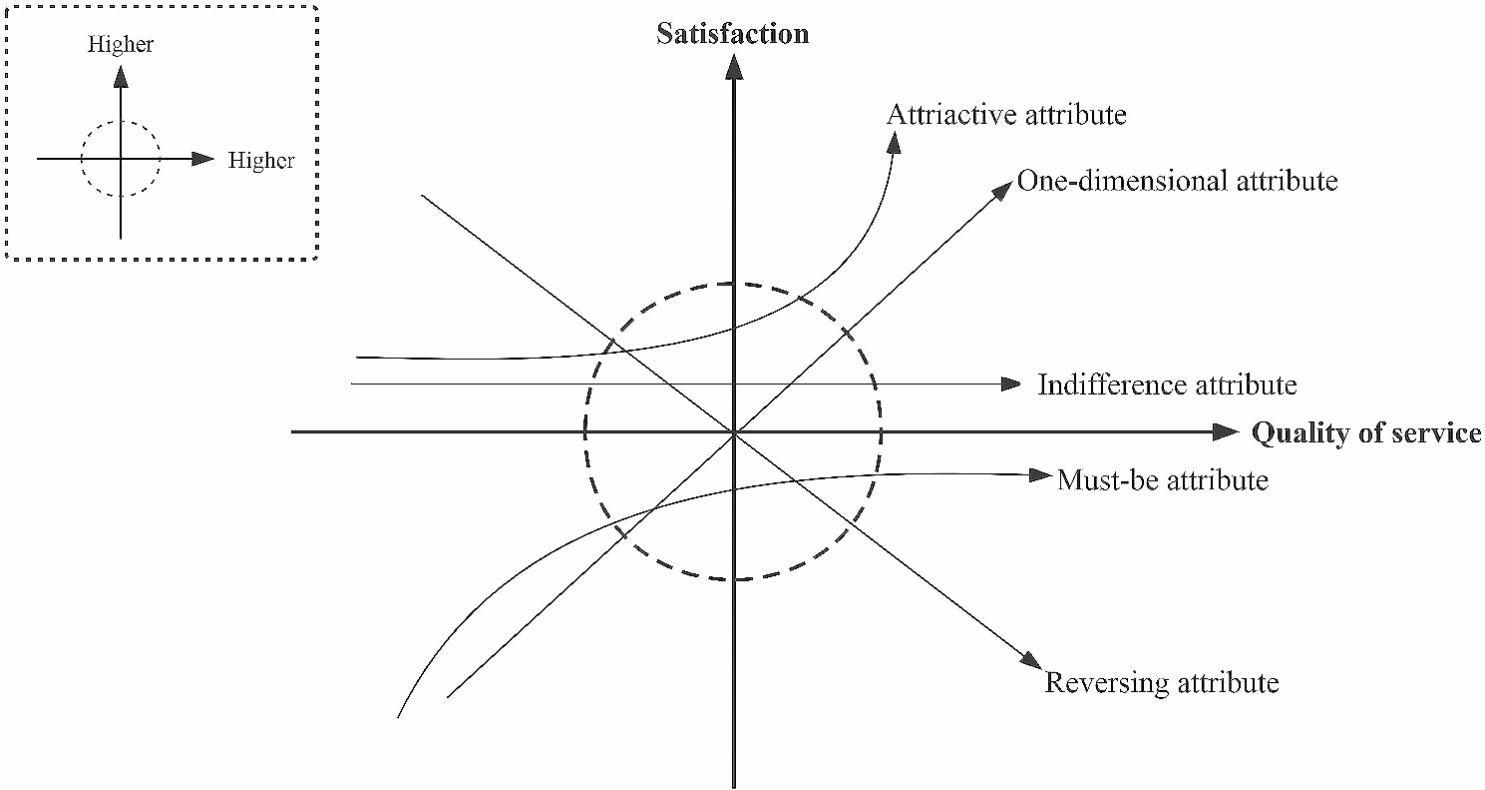
The diagram of correlations between six attributes, satisfaction and quality of service of Kano model

Furthermore, the Kano model is a straightforward analytical tool that may pinpoint the attributes of individual service needs [28]. The Kano model has been extensively utilized to assess patient needs for medical and nursing services as the healthcare industry grows [29]. The model divides the attributes of service needs among patients into six categories: must-be attribute (M), one-dimensional attribute (O), attractive attribute (A), indifference attribute (I), reversing attribute (R), and questioned answer (Q) are created by the model to categorize the characteristics of patients’ service demands. The Kano model diagram showed correlations between six attributes, satisfaction and quality of service, displayed in Fig. 1. The techniques for data collection for the Kano model as follows: when gathering data, the Kano model calls for asking respondents between forward and reversing questions. Respondents were given five options for answering each forward and reversing question. The attributes were categorized as indicated in Table 1. Additionally, the techniques for analyzing data in the Kano model as follows: the attributes were qualitatively defined, sorted, classified, and optimized by applying the Maximum-Frequency analysis model, the Importance-Satisfaction Matrix analysis model (IPA), and the Blue-Sea Strategy analysis model.

**Table 1:** The attributes of spiritual care needs categorizations and data collection methods of the Kano model

Forward questions (If a spiritual care service can be provided in the hospital, what do you think?)	Reversing questions (If a spiritual care service can not be provided in the hospital, what do you think?)				
	Like	Should be	It doesn’t matter	Bearable	Dislike
Like	*Q*	*A*	*A*	*A*	*O*
Should be	*R*	*I*	*I*	*I*	*M*
It doesn’t matter	*R*	*I*	*I*	*I*	*M*
Bearable	*R*	*I*	*I*	*I*	*M*
Dislike	*R*	*R*	*R*	*R*	*Q*

Note:

M: must-be attribute; O: one-dimensional attribute; A: attractive attribute; I: indifference attribute; R: reversing attribute; Q: questioned answer

The specific application methods of the techniques for analyzing data in the Kano model as follows: (1) The Maximum-Frequency analysis model: To specify and categorize attributes. In other words, an attribute is the Kano attribute for an item if it has the highest frequency among the six categories of attributes: O, M, A, I, R, and Q; (2) The Importance-Satisfaction Matrix analysis model (IPA): To determine, analyze, and categorize the significance and level of satisfaction of attributes. The dissatisfaction coefficient after elimination is called the importance (DSI), and the closer it is to 100%, the more of an impact the service has on the importance among patients. The satisfaction (SI) refers to the increased satisfaction coefficient, the stronger the influence of the service on patient satisfaction, the closer the satisfaction (SI) is to 100%. The following are the importance and satisfaction calculation formulas: DSI = (M + O) / (A + M + O + I) and SI = (A + O) / (A + M + O + I) are equal. Using the DSI as the vertical axis and the SI as the horizontal axis, a quadrant chart was created based on the DSI and SI. It was divided into four areas: Predominance Area I, Improving Area II, Secondary Improving Area III, and Reserving Area IV; (3) The Blue-Sea Strategy analysis model: To optimize and analyze the attributes. In other words, consolidating must-be and one-dimensional attributes, optimizing attractive attributes and transforming the indifference attributes. What’s more, it is essential to improve patient service happiness and quality of nursing service that the Kano model was successfully integrated with clinical nursing services, as demonstrated by the study’s findings [30].

To sum up, several previous studies have confirmed the importance of spiritual care needs among patients with cancer. Nevertheless, there were few studies conducted in China on the spiritual care needs among inpatients with advanced breast cancer, and the majority of them were qualitative. Furthermore, the Kano model was hardly ever applied to investigate spiritual care needs. Thus, this study aimed to investigate the spiritual care needs and attributes characteristics of inpatients with advanced breast cancer using the Kano model.

## Objectives

This study aimed to (1) investigate the spiritual care needs score quantitatively among inpatients with advanced breast cancer; (2) to analyze the spiritual care needs attributes qualitatively and define, sort, categorize, and optimize it by the Kano model; and (3) to provide a theoretical foundation for precisely identifying the breakthrough point of improving spiritual care satisfaction, developing focused spiritual care intervention measures, and enhancing spiritual care quality and satisfaction.

## Methods

### Study design and setting

This study, which was carried out in China, used a descriptive cross-sectional design. Additionally, the high-quality reporting of the study adhered to the Strengthening the Reporting of Observational Studies in Epidemiology Statement, which provides guidelines for reporting observational studies and should be included in publications of cross-sectional studies.

### Participants and sample size

From October 2022 to May 2023, Chinese inpatients with advanced breast cancer in the oncology departments of three tertiary grade-A hospitals were the subject of the convenience sample study. The following are the criteria for inclusion and exclusion of the study participants. Inclusion criteria: (1) Inpatients who have been diagnosed with advanced breast cancer based on pathological examination, and follow the diagnostic criteria of advanced breast cancer in the guidelines for clinical diagnosis and treatment of advanced breast cancer in China (2022 edition) [31]; (2) Age ≥ 18 years old; (3) The ability to accurately comprehend and complete the questionnaire and interact with researchers; (4) Voluntary participation. Exclusion criteria: (1) Investigation is precluded by complicated significant diseases (e.g., renal failure, mental illness, etc.); (2) Study participation is or has been recent.

As per the descriptive study by Kendall [32], the approximate sample size estimation method is as follows: *N* = *n* (5–10) × (1 + 10%) and the Hulland et al. [33] determination method on the Kano model questionnaire [*N* ≥ *n* × 10, and ≥ 200]. Since there were 24 items in the study, the sample size required to represent the real hospital setting must be at least *N* = (24 × 5) × (1 + 10%) = 132. The 369 participants were delivered; however, five patients declined to participate in the survey because of their disease. In the end, this study had 357 participants. “*N*” represents the sample size, and “*n*” represents the number of items of questionnaires.

### Measures and variables

#### The demographic characteristics questionnaire

Based on the literature review findings, the researchers developed a questionnaire on demographic and individual characteristics and patients’ socioeconomic characteristics. The questionnaire consisted of 14 items and included information on age, nationality, religious beliefs, marital status, education level, place of residence, residence status, occupation status, monthly income per capita, medical payment methods, disease staging, time to diagnosis, number of hospital admissions, and treatment.

#### The nurse spiritual therapeutics scale (NSTS)

Taylor et al. [34] and Xie et al. [35] developed the NSTS. Cronbach’s α was 0.792, compared to the 0.908 value of this study. It also included 12 items spread across 5 dimensions, including the following: “share self-perception” (5 items), “help thinking” (3 items), “create a good atmosphere” (2 items), “explore spiritual beliefs” (1 item), and “help religious practice” (1 item). Likert 4 scoring system was applied; the scores for “strongly disagree,” “disagree,” “agree,” and “strongly agree” were 1, 2, 3, and 4, respectively. The NSTS has a total score ranging from 12 to 48 points. Scores of 12–24 for mild, 25–36 for moderate, and 37–48 for severe correlate to these categories. Higher scores imply a greater need for spiritual care.

#### The Kano model-based nurse spiritual therapeutics attributes Scale (K-NSTAs)

The K-NSTAs scale was developed by researchers using the NSTS scale and the Kano model, which is used to examine the spiritual care needs of inpatients with advanced breast cancer. The 12 items of NSTS were asked forward and reverse questions based on the Kano model, totaling 24 questions that comprised the K-NSTAs scale, and the expert consultation was carried out. Thirty inpatients with advanced breast cancer were pre-investigated before the official investigation began. The early survey results indicated that forward and reverse questionnaires had Cronbach’s α values of 0.902 and 0.913, respectively.

### Data collection

Participants were enrolled from October 2022 to May 2023 in the oncology departments of three Chinese tertiary grade-A hospitals. First, the researchers are assisted by chief nurse in conducting the inquiry with the prior consent of the hospital management, using the department as the unit. Second, before the investigation, the researchers informed the patients and their families orally face-to-face about the study’s aim, importance, and privacy. The study participants were free to decline or leave at any time, and the information collected will only be used for academic research and not for profit. In this manner, the informed consent form was signed before the research, and the participant’s consent was acquired. When patients were unable to finish on their own, researchers or their families can help them fill out the questionnaire according to according to their own choices by reading the questions to them. In-person, the surveys were face-to-face, anonymous, and completed in around 15–20 min, after which they were immediately collected and verified. In this study, 369 paper questionnaires were delivered; however, five patients declined to participate in the survey because of their disease. A total of 357 valid questionnaires with an effective recovery rate of 96.7% were obtained after removing seven unqualified questionnaires.

### Statistical analysis

Two researchers used Epidata 3.1 software to record and verify the data, while IBM Statistical Package for Social Sciences 21.0 was used for data analysis. Demographic features were described using descriptive statistics (numbers, percentage distribution). Measurement results that fit a normal distribution were expressed as mean ± standard deviation, and a one-way analysis of variance or a two-independent sample *t*-test was used to compare the results between the groups. When describing non-normally distributed measurement data, the median and interquartile range were used; when comparing groups, the Mann-Whitney U test or the Kruskal-Wallis test were used. The Maximum-Frequency analysis model, the Importance-Satisfaction Matrix analysis mode (IPA), and the Blue-Sea Strategy analysis model were employed to define, sort, categorize, and optimize attributes qualitatively.

## Results

### Demographic characteristics

This study comprised 357 inpatients with advanced breast cancer, ages ranging from 25 to 78, with an average age of 42.51 ± 13.87. To sum up, 256 (71.7%) of age were < 60 years old, and 101 (28.3%) of age were ≥ 60 years old. Furthermore, 328 (91.8%) of nationality were Han nationality, 312 (87.4%) had no religious beliefs, 283 (79.3%) of marital status were married, 129 (36.2%) of education level were junior school, 145 (40.5%) resided in cities, and 291 (84.1%) lived with others. And Table 2 displayed further demographic data.

**Table 2:** Demographic characteristics among Chinese inpatients with advanced breast cancer (*n* = 357)

Characteristics	n	%
**Age (years)**		
< 60	256	71.7
≥ 60	101	28.3
**Nationality**		
Han	328	91.8
Minority	29	8.2
**Religion beliefs**		
Yes	45	12.6
No	312	87.4
**Marital status**		
Unmarried	14	3.9
Married	283	79.3
Divorced	38	10.7
Widowed	22	6.1
**Education level**		
Primary school and below	69	19.3
Junior school	129	36.2
High school / Secondary school	102	28.7
Junior college and above	57	16.8
**Place of residence**		
Cities	145	40.5
Towns	99	27.8
Rural area	113	31.7
**Residence status**		
Living alone	66	18.6
Living with others	291	84.1
**Occupation status**		
Be on the job	221	61.8
Not on the job	136	38.2
**Monthly income per capita (RMB)**		
<1000	48	13.4
1000～<3000	106	29.8
3000～<5000	129	36.2
≥ 5000	74	20.6
**Medical payment methods**		
Urban employee medical insurance	186	52.1
Urban and rural residents medical insurance	163	45.6
Others	8	2.3
**Disease staging**		
I	125	35.1
II	183	51.3
III	49	13.6
**Time to diagnosis (months)**		
≤ 1	199	55.8
2～5	124	34.6
>5	34	9.6
**Number of hospital admissions**		
≤ 1	219	61.3
2～5	105	29.5
>5	33	9.2
**Treatment**		
Operation	112	31.5
Chemotherapy	47	13.3
Operation and chemotherapy	174	48.7
Operation and radiation therapy	9	2.4
Operation, chemotherapy and radiation therapy	15	4.1

### The scores of spiritual care needs

The 357 inpatients with advanced breast cancer had a total score of 31.16 ± 7.85 for spiritual care needs, and the average NSTS score was 2.60 ± 0.79. Out of the five dimensions, “create a good atmosphere” had the highest average score (3.16 ± 0.95), while “help religious practice” had the lowest (1.72 ± 0.73). Table 3 displays the average scores for the following three aspects, which were, in order of high to low, “share self-perception” (2.69 ± 0.98), “help thinking” (2.54 ± 0.88), and “explore spiritual beliefs” (2.08 ± 0.65).

**Table 3:** The scores of spiritual care needs among Chinese inpatients with advanced breast cancer [*n* = 357, (M ± SD)]

Dimensions	Items	Average of items		Ranking	Number of items	Dimensions score		Average of dimensions		Ranking
		M	SD			M	SD	M	SD	
NSTS total score	**—**	**—**		**—**	12	31.16	7.85	2.60	0.79	**—**
Share self-perception	Q1. Listen to me talking about my spiritual strengths.	2.89	0.71	3	5	13.43	3.25	2.69	0.98	2
	Q2. Listen to me talking about my spiritual concerns.	2.81	0.69	4						
	Q3. Help me to think about my dreams.	2.58	0.77	7						
	Q4. Teach me about ways to draw or write about my spirituality.	2.45	0.71	10						
	Q5. Listen to the stories of my life.	2.70	0.69	5						
Help thinking	Q6. Ask me about religious practices.	2.47	0.66	9	3	7.62	2.05	2.54	0.88	3
	Q7. Offer to talk with me about meditation or.	2.52	0.70	8						
	Q8. Ask me about what gives my life meaning.	2.63	0.72	6						
Create a good atmosphere	Q9. Bring me humorous things, e.g.: share a joke.	3.04	0.71	2	2	6.31	1.58	3.16	0.95	1
	Q10. Help me to enjoy quiet times or space.	3.27	0.75	1						
Explore spiritual beliefs	Q11. Ask me about my spiritual beliefs.	2.08	0.65	11	1	2.08	0.65	2.08	0.65	4
Help religious practice	Q12. Help me, if I needed, with my religious practices.	1.72	0.73	12	1	1.72	0.73	1.72	0.73	5

### The attributes of spiritual care needs

**Fig. 2:**
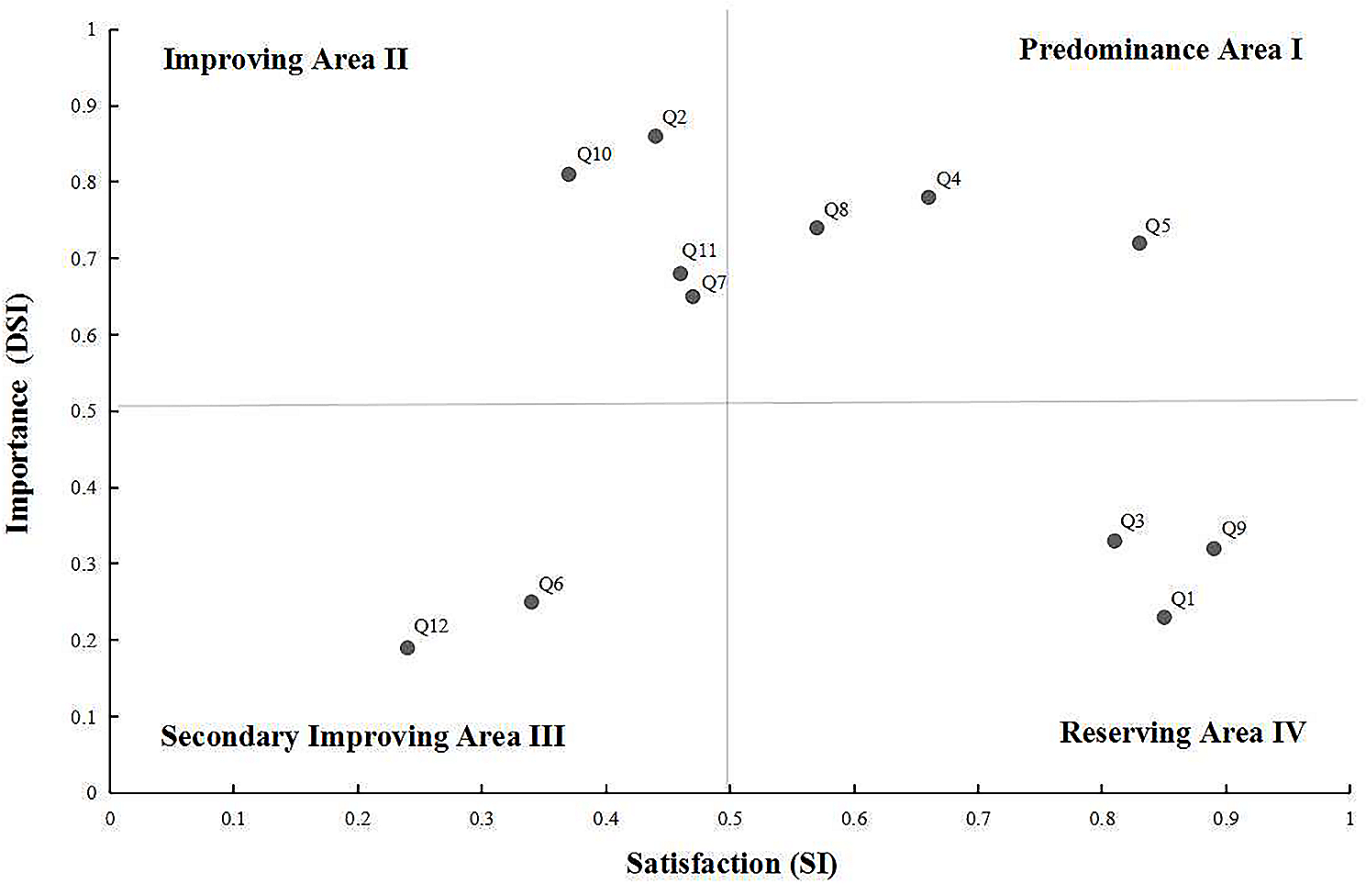
The matrix diagram of spiritual care needs attributes based on the Importance-Satisfaction Matrix analysis model

The 12 items of spiritual care needs were distributed as follows: three attractive attributes (item Q1, Q3, and Q5) were located in Reserving Area IV; five one-dimensional attributes (item Q4, Q5, Q7, Q8, and Q11) were located in Reserving Area IV, three of the five items were located in Predominance Area I (item Q4, Q5, and Q8), and the other two were located in Improving Area II (item Q7 and Q11); two of must-be attributes were located in Secondary Improving Area III (item Q6 and Q12); two of the indifference attributes were located in Improving Area II (item Q2 and Q10). The DSI, SI, and matrix diagram of spiritual care needs attributes were shown in Table 4; Fig. 2, respectively.

**Table 4:** The attributes of spiritual care needs among Chinese inpatients with advanced breast cancer based on the Kano model (*n* = 357)

Dimensions	Items	Composition proportions of attributes based on Kano model (n)						Kano attributes	Satisfaction (SI)	Importance (DSI)
		A	M	O	I	R	Q			
Share self-perception	Q1. Listen to me talk about my spiritual strengths.	263	42	40	8	3	1	A	0.85	0.23
	Q2. Listen to me talk about my spiritual concerns.	34	184	123	14	1	1	M	0.44	0.86
	Q3. Help me to think about my dreams.	237	66	52	2	0	0	A	0.81	0.33
	Q4. Teach me about ways to draw or write about my spirituality.	71	113	165	6	1	1	O	0.66	0.78
	Q5. Listen to the stories of my life.	95	56	201	3	1	1	O	0.83	0.72
Help thinking	Q6. Ask me about religious practices.	73	41	48	193	2	0	I	0.34	0.25
	Q7. Offer to talk with me about meditation or.	38	103	129	83	3	1	O	0.47	0.65
	Q8. Ask me about what gives my life meaning.	54	115	149	36	2	1	O	0.57	0.74
Create a good atmosphere	Q9. Bring me humorous things, e.g.: share a joke.	223	19	95	17	2	1	A	0.89	0.32
	Q10. Help me to have quiet times or space.	61	218	71	6	1	0	M	0.37	0.81
Explore spiritual beliefs	Q11. Ask me about my spiritual beliefs.	38	117	126	74	2	0	O	0.46	0.68
Help religious practice	Q12. Help me, if I needed, with my religious practices.	71	53	15	217	1	0	I	0.24	0.19

Additionally, Table 4 also demonstrated that the “share self-perception” dimension had two attractive attributes (item Q1 and Q3), two one-dimensional attributes (item Q4 and Q5), and one must-be attribute (item Q2). What’s more, there were one indifference attribute (item Q6) and two one-dimensional attributes (item Q7 and Q8) belonging to the “help thinking” dimension. One attractive attribute (item Q9) and one must-be attribute (item Q10) were the “create a good atmosphere” dimension. Moreover, there was a one-dimensional attribute (item Q11) that belonged to the “explore spiritual beliefs” dimension and a attribute that was indifference attribute (item Q12), which belonged to the “help religious practice” dimension.

## Discussion

### The scores status quo of spiritual care needs

In this study, the total score for spiritual care needs was 31.16 ± 7.85 among inpatients with advanced breast cancer. When compared to the findings of Wang et al. [36], the inpatients with advanced breast cancer had intermediate spiritual care demands, meaning that their spiritual care needs need to be further improved. The outcomes of Ayik et al. [37] and Wang et al. [38] were comparable to this. The following could be the cause: (1) Patients with advanced breast cancer are more prone to contemplate and confront mortality because of the adverse reactions of chemotherapy and radiation, which have also diminished their physical function and caused recurrent illnesses. Patients in the later stages experience more significant spiritual troubles and pains than those in earlier stages [7]. They also express a greater desire for support from family and friends, hope to overcome the pain, and seek spiritual strength and comfort to find the confidence and courage to continue treatment, which in turn encourages them to generate spiritual care needs [39]; (2) The spiritual care model in China is still in its infancy [40]. The established clinical care model now in use ignores spiritual care in favor of superficial form and operation technology. Furthermore, China lacks a structured and standardized education model for spiritual care instruction. Patients’ spiritual care needs are not met because nurses have low cognitive levels of spiritual care, limited spiritual care abilities, and inadequate attention to patients’ inner thoughts and needs [41]. The factors mentioned above contribute to the moderate spiritual care needs of inpatients with advanced breast cancer, which still require improvement.

“create a good atmosphere” received the highest average scores out of the five dimensions, which was in line with Nixon’s findings [42], which noted that many patients have a strong desire for solitude and would like to have their own space where they can feel comfortable and at peace with themselves. The explanation could be that the majority of inpatients with advanced breast cancer have poor physical function, psychological quality, and social skills and relatively calm and empty hearts. They hope that nurses will be able to give them a quiet, peaceful place to be throughout their illness and provide them with humor and jokes to cheer them up [4, 5]. Furthermore, they hope that nurses will be open to sharing their life stories, spiritual struggles, and triumphs and growing in confidence to understand the significance of life and death. This will enable them to overcome negative emotions like inner anxiety and fear, stay upbeat, and search for the meaning and purpose of life [10]. The factors mentioned above, therefore, contributed to the highest score of this dimension. As a result, nurses ought to provide patients with personal space, foster a caring and welcoming ward atmosphere, and do everything within their power to assist their bodies, minds, societies, and spiritualities.

Furthermore, “help religious practice” had the lowest average scores across all dimensions. More than three-quarters of the patients in this study did not hold any religious beliefs. Additionally, China is a socialist country where the vast majority of people adhere to materialism and atheism. As a result, religion and beliefs are not as strong in China as they are in Eastern culture, even though most people respect China’s belief in red culture [43]. As a result, most patients are opposed to and resistant to religious practices and rituals. For the reasons listed above, this dimension had the lowest score. Thus, to meet their patients’ spiritual care needs, nurses should focus on material directly tied to religious belief. Spiritual care can incorporate religious and traditional Chinese philosophy.

### The attributes status quo of spiritual care needs

#### Must-be and one-dimensional attributes of spiritual care needs should be consolidated

The most fundamental attribute of patients is a must-be attribute, defined by its strong impact on the significance of the patients’ service needs but its minimal impact on satisfaction. Research has validated that it ought to be attended to prioritizing the demands of patients. The importance and satisfaction of patients are greatly influenced by the one-dimensional attribute, which also plays a crucial role in enhancing the standard and contentment of spiritual care provided in hospitals [44]. The findings of this study demonstrated that among inpatients with advanced breast cancer, “share self-perception,” “create a good atmosphere,” and “help thinking” dimensions accounted for the majority of must-be and one-dimensional traits. This could be because the physical, psychological, and social burdens and psychological endurance of older in patients with advanced breast cancer are not as significant as those of younger and middle-aged inpatients during the treatment and prognosis stages of the disease [37]. As a result, people are more willing to communicate with their family, friends, and nurses to express their emotions and discover the purpose and worth of life as they near death. Currently, though, their ability to communicate with family and friends while illness is restricted due to the medical environment, disease treatment, and other factors. Thus, they are unable to communicate with them while they are in the hospital; instead, they must rely on the nurses to offer appropriate assistance and support (such as a joke and a quiet and lonely environment, etc.) to help them express their anxiety and pains, reflect on their past experiences, maintain a positive outlook, affirm their existence, and pursue spiritual peace [45]. Therefore, the factors mentioned above led to must-be and one-dimensional attributes, with a focus on the dimensions of “share self-perception,” “create a good atmosphere,” and “help thinking.”

Consequently, it is recommended that nursing administrators prioritize the needs of “share self-perception,” “create a good atmosphere,” and “help thinking” dimensions and that must-be and one-dimensional attributes be consolidated following the unique characteristics of patients. To help patients feel more at ease spiritually, nurses should provide them with a quiet and lonely environment, practice active listening, actively communicate with patients by using appropriate speech techniques, and listen to their spiritual problems, pains, life stories, and experiences through listening, companionship, and empathy.

#### Attractive attributes of spiritual care needs should be optimized

The primary feature of the significant development of the hospital, the attractive attribute, is a type of attribute for patients’ surprise. It is distinguished by its minimal influence on the importance of patients but its strong influence on satisfaction. According to a study, the patient’s contentment would increase significantly if their charm attributes can be fully met [45]. The findings of this study demonstrated that attractive attributes among inpatients with advanced breast cancer were mainly concentrated on “share self-perception” and “create a good atmosphere” dimensions. The following could be the cause: during treatment and prognosis, patients with advanced breast cancer may experience a gradual decline in their physical function, psychological quality, and social ability as a result of surgery, chemotherapy, and radiotherapy. Additionally, they may experience an increase in the burden of self-feeling, which may lead to a range of negative emotions, including anxiety, depression, inferiority, fear, and even suicidal thoughts [10]. As a result, to effectively confront disease, family, and life, they desperately require external support and assistance to validate the worth of self-existence and discover the meaning and purpose of life [6]. According to the findings of Li et al. [46], the majority of inpatients with advanced breast cancer also want to connect with positive and optimistic people and things while undergoing treatment and learning about their prognosis. They want to receive support, affirmation, encouragement, and empathy from family, friends, and nurses and boost their confidence in continuing their course of treatment. Thus, “share self-perception” and “create a good atmosphere” dimensions constituted the main focus of the above causes, leading to attractive characteristics.

Consequently, it is recommended that nursing administrators focus on “share self-perception” and “create a good atmosphere” and that the attractive attributes of spiritual care need to be optimized. To help patients regain their significance for disease treatment and survival, nurses should actively support and help them solve existing problems as much as possible. This will allow patients to talk about their inner troubles and pains, encourage them to share their experiences and receive more positive feedback when providing spiritual care.

#### Indifference attributes of spiritual care needs should be transformed

An indifferent attribute has minimal influence on the significance and contentment of patients. It is no longer a necessary attribute for hospitals to improve nursing quality, according to a study. The findings of this study demonstrated that the “help thinking” and “help religious practice” dimensions accounted for the majority of indifferent traits among inpatients with advanced breast cancer. The following could be the leading causes of this: only about 10% of the patients in this study identified as religious, and the majority of patients who do not identify as spiritual feel repulsed by them. Thus, this could be a significant contributing factor to the conclusion, which was consistent with the findings of Zhang [47]. Furthermore, the religious-cultural atmosphere in China is weaker than that of Western nations in comparison to their spiritual cultures. Additionally, the majority of patients in China do not practice any religion because of external factors like the traditional culture in China. As a result, many religious practices (including offering sacrifices, praying, chanting, worshipping, etc.) cannot be effectively carried out in China [48]. Nonetheless, research has demonstrated that religious belief is a vital component of spiritual care needs and a means of expressing spirituality in and of itself. Patients who practice religion have better inner serenity [43, 49]. Thus, the factors listed above contribute to indifference mainly focused on the “help thinking” and “help religious practice” dimensions.

Consequently, it is recommended that nursing administrators adjust their apathy toward spiritual care needs according to patients’ unique characteristics and cultural backgrounds. Nurses should pay particular attention to topics about religious beliefs and provide spiritual care tailored to their specific needs, mainly if they practice a particular religion. Additionally, nurses can enhance their sense of self-existence, dignity, and worth and obtain spiritual comfort by using dignity treatment, painting therapy, meaning therapy, and other techniques.

### Limitations

The study was nonetheless subject to the following limitations: (1) First, the study used a convenience sampling method, and only 357 Chinese inpatients with advanced breast cancer were enrolled in three hospitals. This may have resulted in unrepresentative samples, somewhat biased results, and no generalization; (2) Second, the study was only cross-sectional quantitative research, lacking qualitative research, longitudinal research, and intervention research to verify the research conclusions further; (3) Finally, because the NSTS is a Chinese version scale, and because Eastern and Western “spirituality” cultures differ, as does the nature of the concept of “spiritual care,” patients may not have a complete understanding of spirituality during the investigation process, which could lead to some bias in the results. Thus, it is recommended that patients with advanced breast cancer from all levels and locations be enrolled and that further studies employ investigation tools and analytical model tools appropriate for Chinese culture.

## Conclusion

The findings of the study indicated that patients with advanced breast cancer had moderate needs for spiritual care, which need to be further improved. Additionally, the items in the “create a good atmosphere” and “share self-perception” dimensions were primarily must-be and one-dimensional attributes, whereas the items of “share self-perception” and “help thinking” dimensions were principally attractive attributes. To improve the standard and satisfaction of spiritual care in hospitals, it is recommended that nursing administrators optimize attractive attributes, transform the indifference attributes based on the consolidation of must-be and one-dimensional attributes, and further develop targeted spiritual care intervention programs.

### Electronic supplementary material

Below is the link to the electronic supplementary material.

Supplementary Material 1

## Data Availability

The relevant data of this study can be obtained from the first author or corresponding author on reasonable request.
